# Temperature, Crystalline Phase and Influence of Substrate Properties in Intense Pulsed Light Sintering of Copper Sulfide Nanoparticle Thin Films

**DOI:** 10.1038/s41598-018-20621-9

**Published:** 2018-02-02

**Authors:** Michael Dexter, Zhongwei Gao, Shalu Bansal, Chih-Hung Chang, Rajiv Malhotra

**Affiliations:** 1 Department of Mechanical and Aerospace Engineering, Rutgers University, Piscataway, New Jersey 08854 USA; 2 Department of Chemical Engineering, Oregon State University, Corvallis, Oregon, 97331 USA; 3 School of Mechanical, Industrial and Manufacturing Engineering, Oregon State University, Corvallis, Oregon, 97331 USA

## Abstract

Intense Pulsed Light sintering (IPL) uses pulsed, visible light to sinter nanoparticles (NPs) into films used in functional devices. While IPL of chalcogenide NPs is demonstrated, there is limited work on prediction of crystalline phase of the film and the impact of optical properties of the substrate. Here we characterize and model the evolution of film temperature and crystalline phase during IPL of chalcogenide copper sulfide NP films on glass. Recrystallization of the film to crystalline covellite and digenite phases occurs at 126 °C and 155 °C respectively within 2–7 seconds. Post-IPL films exhibit p-type behavior, lower resistivity (~10^−3^–10^−4^ Ω-cm), similar visible transmission and lower near-infrared transmission as compared to the as-deposited film. A thermal model is experimentally validated, and extended by combining it with a thermodynamic approach for crystal phase prediction and via incorporating the influence of film transmittivity and optical properties of the substrate on heating during IPL. The model is used to show the need to a-priori control IPL parameters to concurrently account for both the thermal and optical properties of the film and substrate in order to obtain a desired crystalline phase during IPL of such thin films on paper and polycarbonate substrates.

## Introduction

Rapid low-temperature sintering of nanoparticles (NPs) into thin films and patterns over large areas is of significant interest for scalable manufacturing of functional devices on rigid and flexible substrates. Compared to existing methods for NP sintering (oven-based sintering, rapid thermal, laser, microwave and electrical) the Intense Pulsed Light Sintering (IPL) process has concurrent advantages of large-area (e.g., ≥12 inches × 0.75 inches here) and high-speed of sintering^1^. IPL uses pulsed, broad-spectrum (350–750 nm) light from a xenon lamp for sintering metallic (Ag^2,3^, Cu^4,5^) and semiconducting chalcogenide (CdS^6^, CdTe^7^, CIGS^8^, CZTS^9^) NPs. Copper Sulfide (Cu_x_S, x = 1 to 2) is an earth-abundant chalcogenide, and is thus cheaper and less toxic than many other chalcogenides (e.g., CdS and CdTe). Cu_x_S thin films have found uses in transistors^10^, switches^11^, Lithium ion batteries^12,13^, electroluminescent devices^14^ and solar control window coatings^15–18^. While Cu_x_S NP thin films have been synthesized using vacuum-based methods^10,19,20^ the solution-based nanoparticle deposition approaches like Chemical Bath Deposition^21^ enable simpler operation, lower cost, and lower temperature of deposition^22^. However, the deposited NPs often require post-deposition sintering to obtain well defined crystal phases and desired optical-electronic properties for the above applications^15,23,24^.

Past work on IPL of metallic NPs has performed experiments and modeling to predict the temperature evolution. Chung *et al*.^25^ monitored conductivity evolution during IPL using a wheatstone bridge to find optimal IPL parameters for silver NPs. This method was extended^26,27^ via measurement of temperature using a thermocouple embedded into the film. A thermal model based on the heat transfer equation, with the film’s optical absorbtance as the heat source and convective and radiative losses to the ambient was validated against experimental temperature evolution. Unlike models of laser sintering, this approach accounts for the broad-spectrum nature of the xenon lamp light. Bansal *et al*.^3^ showed a self-limiting behavior during IPL of Ag NPs due to progressive reduction in optical absorption with increasing densification, resulting in a turning point in temperature evolution during IPL.

Past work on IPL of CdS^6,28^ has shown grain growth due to fusion and sintering of the NPs and evaporation of Sulfur from the as-deposited film. However, no change in crystal phase was observed after IPL. While an increase in crystallinity was observed, no change in crystal structure was seen in IPL of CdTe NP films^29^ or perovskite NP films^30,31^. IPL was applied to a CIG metallic alloy and Se NP composite film to fabricate Cu(In,Ga)Se_2_ thin films via a NP melting based alloying approach^32^, involving a physical phase change. Modeling of temperature evolution during IPL of CdS NP films on glass^6^ has used a similar approach as above to predict temperature evolution as a function of IPL parameters.

In these works, while crystallite size increases due to IPL there is little or no change in crystal structure, which obviates the need for crystal phase prediction. Chalcogenides like Cu_x_S have multiple polymorphs and can change crystal phase (as shown in this work) during IPL, along with a corresponding change in the film properties^15,23,24^. Further, the above models do not consider xenon light transmitted through the film as a source of heat at the film-substrate interface. If the film is thin enough and has appreciable transmission (as is the case here), and uses an opaque or translucent substrate (e.g. paper), then this heat source can influence temperature rise and crystal phase change in the film. Also, the presence or absence of the self-limiting behavior seen in IPL of metal NPs has yet to be confirmed in IPL of chalcogenides. The model used to predict temperature evolution needs to be different if there is coupling between the crystal phase and NP densification and optical absorption^3,33^, as compared to the conventional thermal model used in literature^6,26^.

This work focusses on experimentally characterizing and modeling the evolution of temperature and phase change, specifically change in crystalline structure of Cu_x_S into different polymorphs, during IPL of Cu_x_S NP thin films. Experimentally measured evolution of film temperature during IPL of the films on glass substrates is correlated to the film’s crystalline phase, morphology, electrical properties and optical properties after IPL. This temperature evolution is also used to observe the presence or absence of self-limiting behavior during IPL. Based on these observations a thermal model is used to model temperature evolution and is extended as follows. First, the evolution of temperature is linked to the change in crystalline phase content of the film via an experimentally derived phenomenological approach for film phase evolution. Secondly, the extended thermal model accounts for the non-negligible transmittivity of the copper sulfide film and the resulting secondary heat source that is created at the film-substrate interface when using opaque substrates like paper. The temperature predictions are quantitatively validated against experimental measurements, and then the extended model is used to understand temperature evolution and crystalline phase change during IPL of Cu_x_S NP films on paper and polycarbonate substrates. The implications of these observations on scalability of IPL of Cu_x_S films and the tailoring of IPL parameters when using substrates with different optical and thermal properties are discussed.

**Figure 1:**
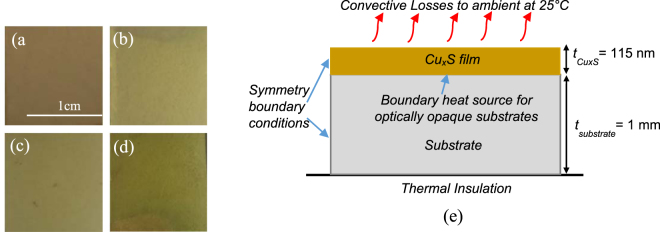
Optical images of (**a**) as-deposited film (**b**) Post-IPL film with fluence E4-2 pulses-0.15% duty cycle (**c**) Post-IPL film with fluence E4-5 pulses-0.15% duty cycle (**d**) Post-IPL film with fluence E4-5 pulses-0.08% duty cycle. (**e**) Schematic of thermal model for IPL. t_CuxS_ and t_substrate_ denote the thickness of the copper sulfide thin film and the substrate respectively.

The experimental and computational approach adopted is briefly described here and is discussed in greater detail in the Methods section. Copper Sulfide NP thin films were deposited on 1 mm thick 2.54 cm × 1.9 cm glass substrates via chemical bath deposition, after Vas-Umnuay *et al*.^21^ (details in Supplementary Discussion S1). The IPL setup consisted of a pulsed xenon flash lamp (Sinteron 3000, Xenon Corporation), and a thermal camera (MicroEpsilon Thermoimager TIM 200, maximum temperature 1500 °C) for measuring film temperature. The response time of this camera was higher (8 ms frame rate) than the on-time of the xenon lamp. To get as close as possible to measuring the peak temperature per pulse the temperature measurement was started well before the flashes from the xenon lamp and the measurement was performed over 5 different samples in a randomized manner. The temperatures shown here are from at least 3 samples that were most consistent with each other, with a standard deviation of no more than 10–15% in peak temperature per pulse. Further, since the off-time is on the order of milliseconds it is likely that the worst error in missing the peak temperature was in the first pulse. The IPL pulse fluence, duty cycle and number of pulses were varied (Table 1). Pulse fluence was varied over 5, 7.5, 10 and 15 J/cm^2^. Since phase change can be a function of heating and cooling time in addition to temperatures during heating and cooling, two pulse duty cycles of 0.08% and 0.15% were used with 5 IPL pulses for each fluence. The change in duty cycles was effected via a constant on-time and changing off-time, to understand the role of cooling time (off time). The irradiance, i.e., (fluence/on time from Table 1) was constant at 6.9 kW/cm^2^. Additional experiments with two IPL pulses at 0.15% duty cycle were also performed for all fluences in Table 1. Representative optical images of as-deposited and post-IPL films are shown in Fig. 1a–d.

**Table 1:** IPL Parameters used in experiments.

Fluence *(J/cm*^2^)	On time-Off time (*ms*)	Duty Cycle (%)	No. of pulses
E1 = 5	0.715–471	0.15	5 (for all duty cycles)
	0.715–942	0.08	
E2 = 7.5	1.075–709	0.15	
	1.075–1418	0.08	
E3 = 10	1.435–947	0.15	2 (for 0.15% duty cycle only)
	1.435–1894	0.08	
E4 = 15	2.150–1419	0.15	
	2.150–2838	0.08

Cross-sectional Scanning Electron Microscopy (SEM) was used to characterize film micromorphology and thickness. Atomic Force Microscopy (AFM), Energy Dispersive Spectroscopy (EDS) and Grazing Incidence X-ray Diffraction (GIXRD) were used to determine roughness, elemental composition and crystal phase of the films respectively. Optical transmittance, reflectance and absorbtance were obtained using a spectrophotometer equipped with an integrating sphere. Sheet resistance was measured using a Signatone four-point probe and was used along with the film thickness to obtain bulk resistivity. Hall effect measurements were used to characterize charge carrier concentration and mobility in the films.

A thermal finite element model was implemented in COMSOL, consisting of a 115 nm thick Cu_x_S film on a 1 mm thick glass substrate (Fig. 1e) with conductive losses allowed between film and substrate. The film thickness and volume were obtained from experimentally measured thickness and deposition area, and shrinkage during IPL was ignored for simplicity, as in past work^26^. The thermal properties of the film and substrate were fixed at average room temperature values obtained from literature (see Supplementary Table S1). The glass substrate was modelled as an infinite element layer in the thickness with the bottom of the substrate being thermally insulated, as in our experiments. Since the surface area of the top of the film is much larger than that of its side walls only convective losses from the top surface of the film were accounted for, and symmetry boundary conditions were used on the side walls of the film-substrate assembly. The optical absorbtance of the film within the energetic spectrum of the xenon lamp, and its evolution during IPL, were experimentally obtained. The predicted and experimentally measured temperature evolution were compared for four cases, namely for 5 pulses at duty cycles 0.15% and 0.08% and fluence E1 and E4. Cases with optically opaque paper and transparent polycarbonate substrates of the same thickness as the glass substrate were also modeled, with appropriate thermal properties of the substrates (see Supplementary Table S1). For opaque paper, xenon light transmitted through the Cu_x_S film will directly heat the paper surface. So, the experimentally observed film transmission was used along with the incident lamp energy and the xenon lamp spectrum to add a boundary heat source at the film-paper interface (Fig. 1e). For the IPL experiments performed on glass substrates a quantitative measure of phase evolution was developed from GIXRD measurements, correlated to the dissipated energy in the film, and used to understand the film phase evolution during IPL on paper and polycarbonate substrates.

## Results

### Experimental

**Figure 2:**
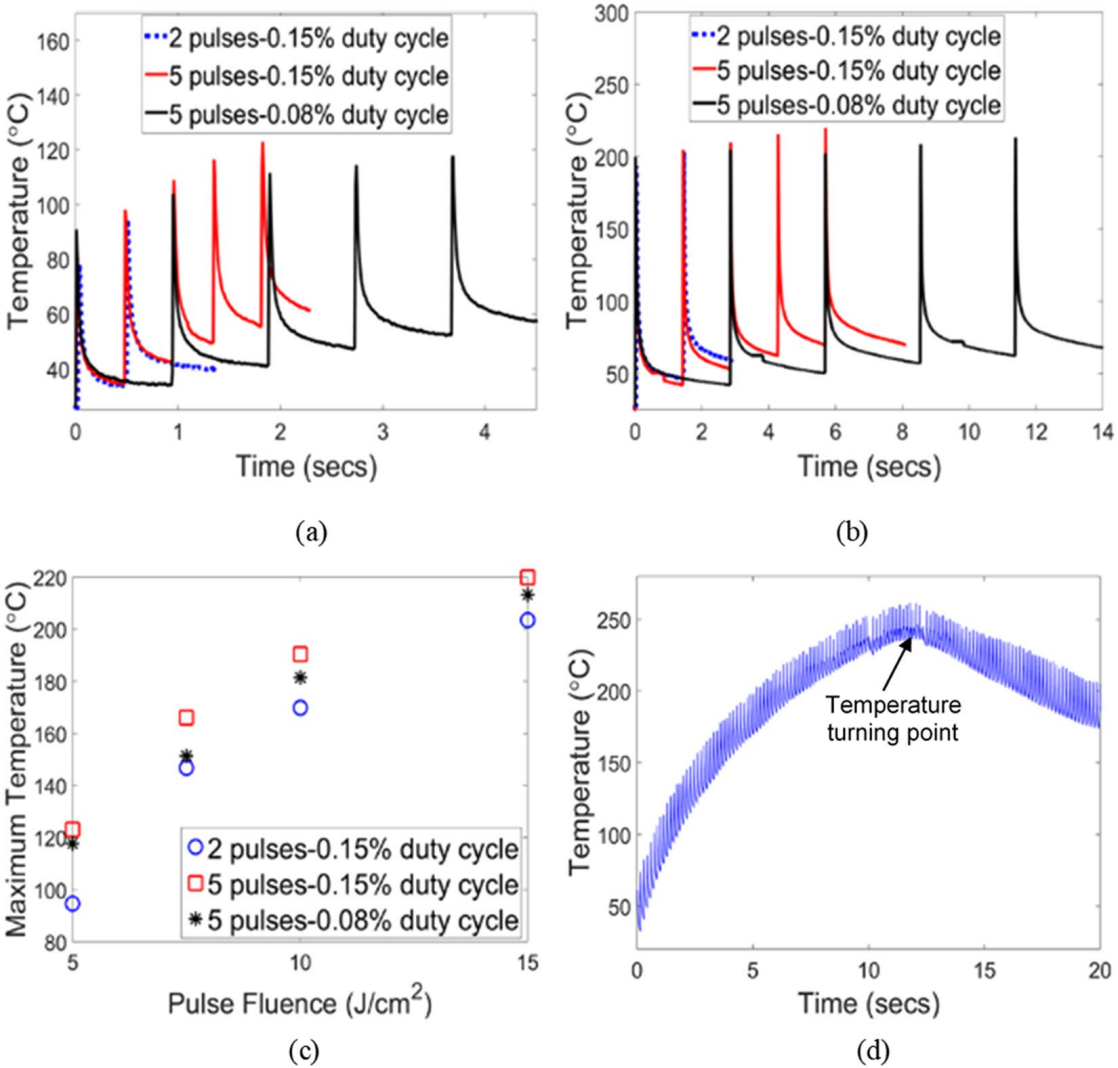
Representative film temperature evolution with different pulse duty cycle and number of pulses and at (**a**) fluence E1 (**b**) fluence E4. (**c**) Maximum film temperature averaged over at least three measurements for varying IPL pulse fluence, number of pulses and duty cycle. Zero pulses and duty cycle represent the unsintered film. Standard deviation in peak temperature measurement was 10–15%. (**d**) Representative example of temperature evolution and temperature turning point during IPL of Ag NPs^3^.

Rise in film temperature during IPL is faster with increasing pulse fluence and duty cycle, and maximum film temperature is greater with greater number of pulses (Fig. 2a–c). The increasing temperature per pulse seen in Fig. 2a,b was also observed for fluences E2 and E3 (see Supplementary Fig. S1). Past work on IPL of metallic silver NPs has shown that there is a turning point in temperature evolution after a certain amount of densification between NPs, and that after this point the peak temperature in each pulse reduces from one pulse to the next (Fig. 2d). It has also been shown that this is due to a progressive reduction in optical absorption by the deposited NPs in the energetic spectrum of the xenon lamp (400–700 nm) with increasing shrinkage and neck growth^3^. Despite significant shrinkage and phase change in the film in our experiments (shown later) there is no observable turning point in film temperature evolution observed here.

**Figure 3:**
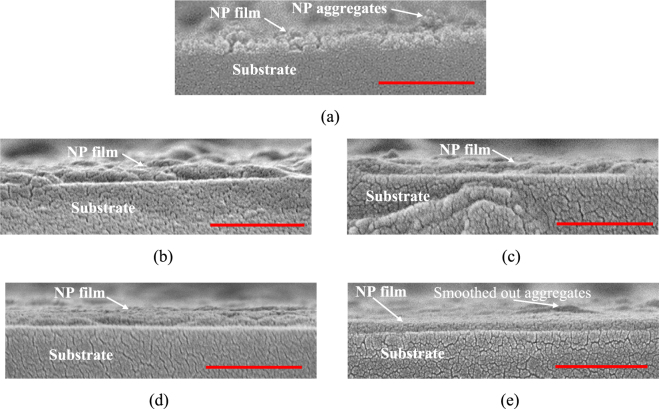
Representative cross-sectional SEM images of (**a**) As-deposited film and post-IPL films at (**b**) Fluence E1 (**c**) Fluence E2 (**d**) Fluence E3 (**e**) Fluence E4. All post-IPL images shown for 5 pulses and 0.15% duty cycle. Red length scales correspond to 500 nm.

**Figure 4:**
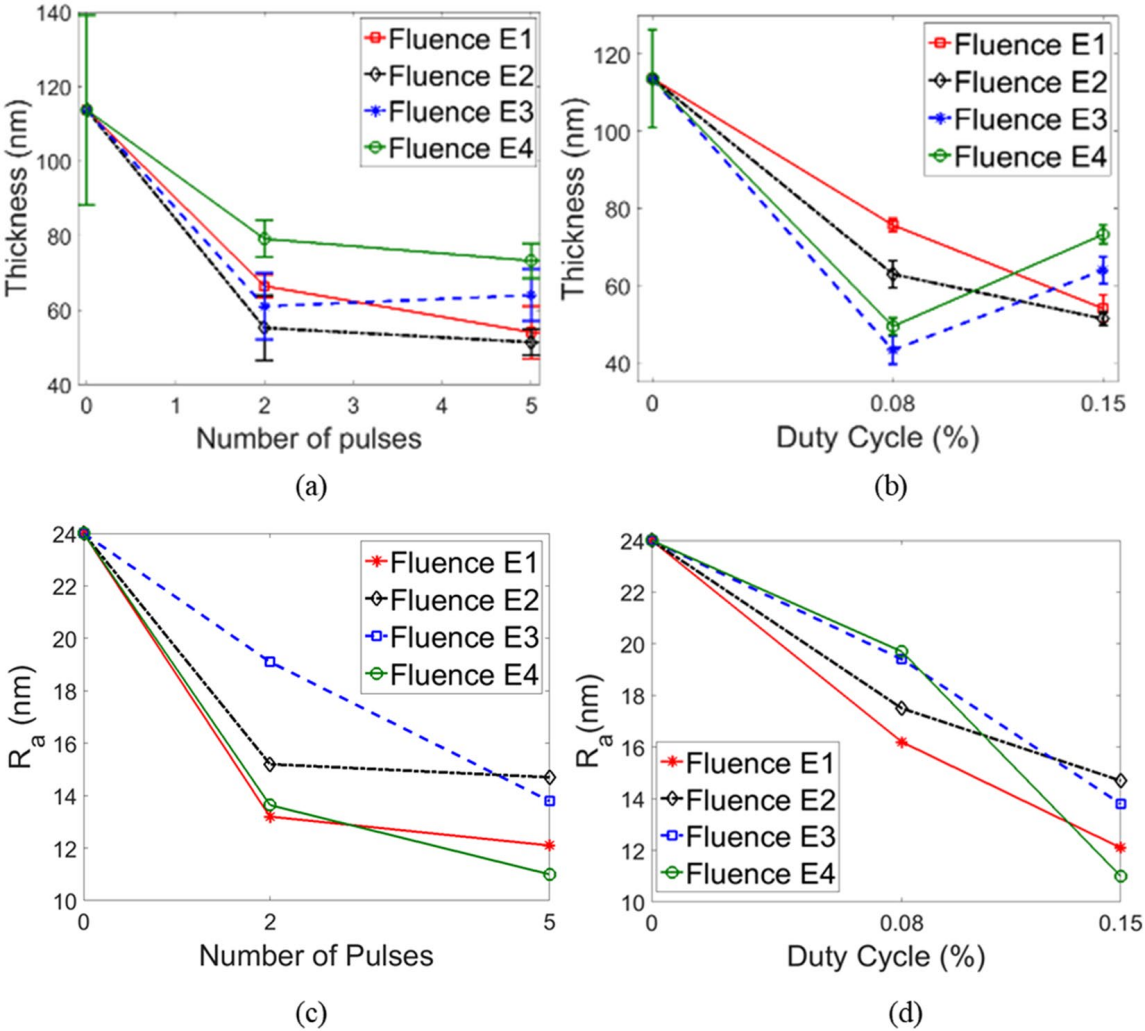
Film thickness for (**a**) varying pulse number and fluence at 0.15% duty cycle (**b**) varying duty cycle and fluence for 5 pulses. Markers show average thickness and error bars show standard deviation over 10 measurements. Average roughness R_a_ for (**c**) varying pulse number and fluence at 0.15% duty cycle (**d**) varying duty cycle and fluence for 5 pulses. Zero pulses and duty cycle represent the unsintered film.

**Figure 5:**
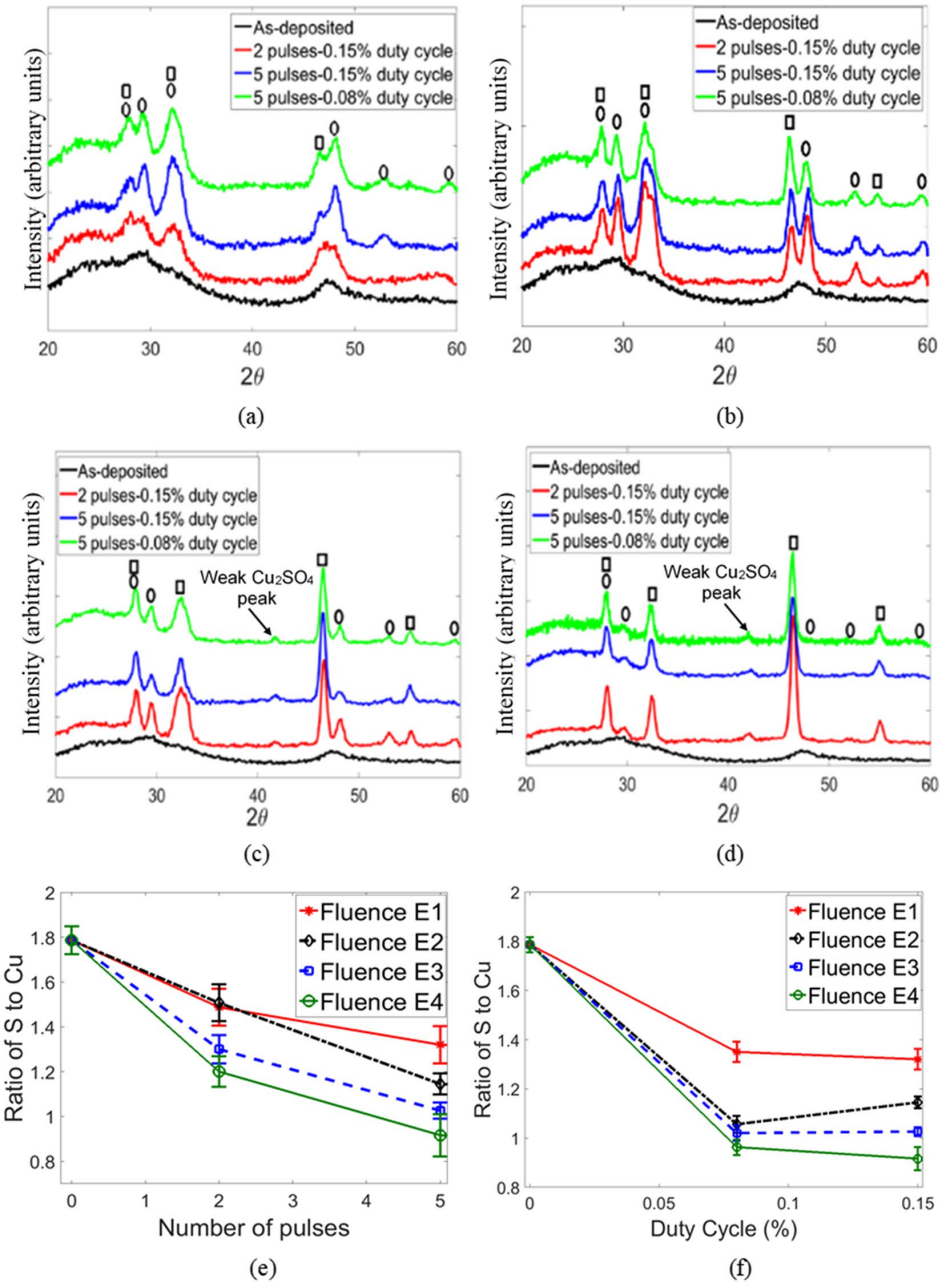
GIXRD spectra for IPL fluence (**a**) E1 (**b**) E2 (**c**) E3 (**d**) E4.  and  represent covellite and digenite peaks respectively. Atomic percentage of S to Cu for various (**e**) number of pulses (**f**) duty cycle. Markers show average ratio of S to Cu and error bars show standard deviation over five measurements for each IPL parameter combination. Zero pulses and duty cycle represent the unsintered film.

SEM images of the unsintered films (Fig. 3a) show individually distinguishable NPs with a rough and bumpy surface due to larger NP aggregates sticking out of the surface of the film. The post-IPL films (Fig. 3b–e) show a significant reduction in individually distinguishable NPs and smoothing out of the NP aggregates, which indicates fusion of the NPs. Figure 4a,b show a reduction in film thickness and surface roughness with increasing pulse number and duty cycle at a given fluence. This is due to evaporation of Sulfur, shrinkage between NPs due to NP fusion, or a combination of both phenomena. The slight increase in film thickness for fluence E3 and E4 at 5 pulses (Fig. 4a) and a more significant increase at 0.15% duty cycle (Fig. 4b) is likely due to the formation of Cu_2_SO_4_, an oxidation by-product during annealing of Cu_x_S films in air, which increases film mass^24,34^. Weak peaks of Cu_2_SO_4_ were observed under these IPL parameters as shown later in Fig. 5c,d. Figure 4c,d show a reduction in average roughness of the film with increasing duty cycle and number of pulses, supporting the observation of NP fusion into a smoother surface due to IPL.

Intensity versus 2*θ* plots from GIXRD (Fig. 5a–d) show that the as-deposited film is largely amorphous, due to the absence of sharp distinguishable peaks. The IPL sintered films show crystalline peaks that can be indexed to hexagonal covellite CuS (JCPDS card 06-0464) and cubic digenite Cu_1.8_S (JCPDS card 24-061).

Some peaks of covellite and digenite at 2θ = 27.75° inherently overlap and some peaks for CuS (major peak 2θ = 31.82°, minor peak 2θ = 32.87°) and Cu_1.8_S (major peak at 2θ = 32.15°) are not easily distinguishable here. However, other peaks for CuS (major peaks 2θ = 29.46°, 48.06°; minor peaks 2θ = 52.84°, 59.56°) and for Cu_1.8_S (major peak 2θ = 46.42°; minor peak 2θ = 55.06°) are clearly distinguishable so that the dominant crystalline phase in the film can be identified. As indicated by the increasing intensity of the 2θ = 48.06° major covellite peak relative to the intensity of the 2θ = 46.42° major digenite peak (Fig. 5a), at low fluence E1 the post-IPL film has a primarily covellite phase and the dominance of the covellite phase increases with increasing number of pulses and duty cycle. At intermediate fluences E2 and E3 (Fig. 5b,c) the digenite peaks start becoming more dominant till at fluence E4 (Fig. 5d) a primarily digenite phase is formed with a near-complete disappearance of the covellite content. Comparing these observations to the maximum film temperature in Fig. 2c, we observe that a crystalline covellite dominant phase is obtained at temperatures as low as 126 °C within 2.3 seconds of IPL (fluence E1, 5 pulses, duty cycle 0.15%) and recrystallization to a digenite-rich phase occurs at temperatures as low as 155 °C within 7.1 seconds of IPL (Fluence E2, 5 pulses, duty cycle 0.08%).

Previous work has reported recrystallization of amorphous Cu_x_S NP films to covellite at 200 °C and to digenite at 250 °C after conventional thermal annealing for an hour^35^. The temperatures and times in which we observe recrystallization of these phases in our IPL experiments are even lower than the above reported values. This is likely due to a combination of the higher specific surface energy of NPs which reduces the temperature needed for evaporation of sulfur atoms from the lattice and consequent rearrangement of the remaining atoms resulting in recrystallization^36^, and rapid localized heating of the film by the xenon lamp light, although the exact contribution of each effect remains to be verified. Within our knowledge, past work on IPL of chalcogenides does not show a change in crystalline phase^6,28–32^, even though a change in crystallite size due to sintering is observed.

Figure 5c,d also show a weak Cu_2_SO_4_ peak at 2θ = 41.48° (JCPDS card number 11-0646), indicating that the oxidation products present are mostly in the amorphous form. This formation of Cu_2_SO_4_ is likely responsible for the increase in thickness seen at fluence E3 and E4 at 0.15% duty cycle and 5 pulses (Fig. 4a,b).

The ratio of atomic percentage of sulfur to copper in the film (Fig. 5e,f), measured via EDS, shows that the as-deposited film has a stoichiometry corresponding to Cu_1.8_S. The overall reduction in S to Cu ratio after IPL indicates the loss of sulfur from the film with increasing fluence, pulse number and duty cycle in IPL, which are concurrent with increasing maximum film temperatures (Fig. 2c). The reduction in the sulfur content during IPL is due to its evaporation from the film, as is seen in conventional annealing of Cu_x_S films in air^18,23,24,34^. Representative EDS spectra for bare glass substrate, as-deposited film and IPL sintered film can be found in Supplementary Fig. S2. Since the as-deposited film is sulfur rich with subsequent evaporation of sulfur and concurrent increase in temperature during IPL, the phase evolution in Fig. 5a–d is in line with the Cu-S phase diagram^37^.

**Figure 6:**
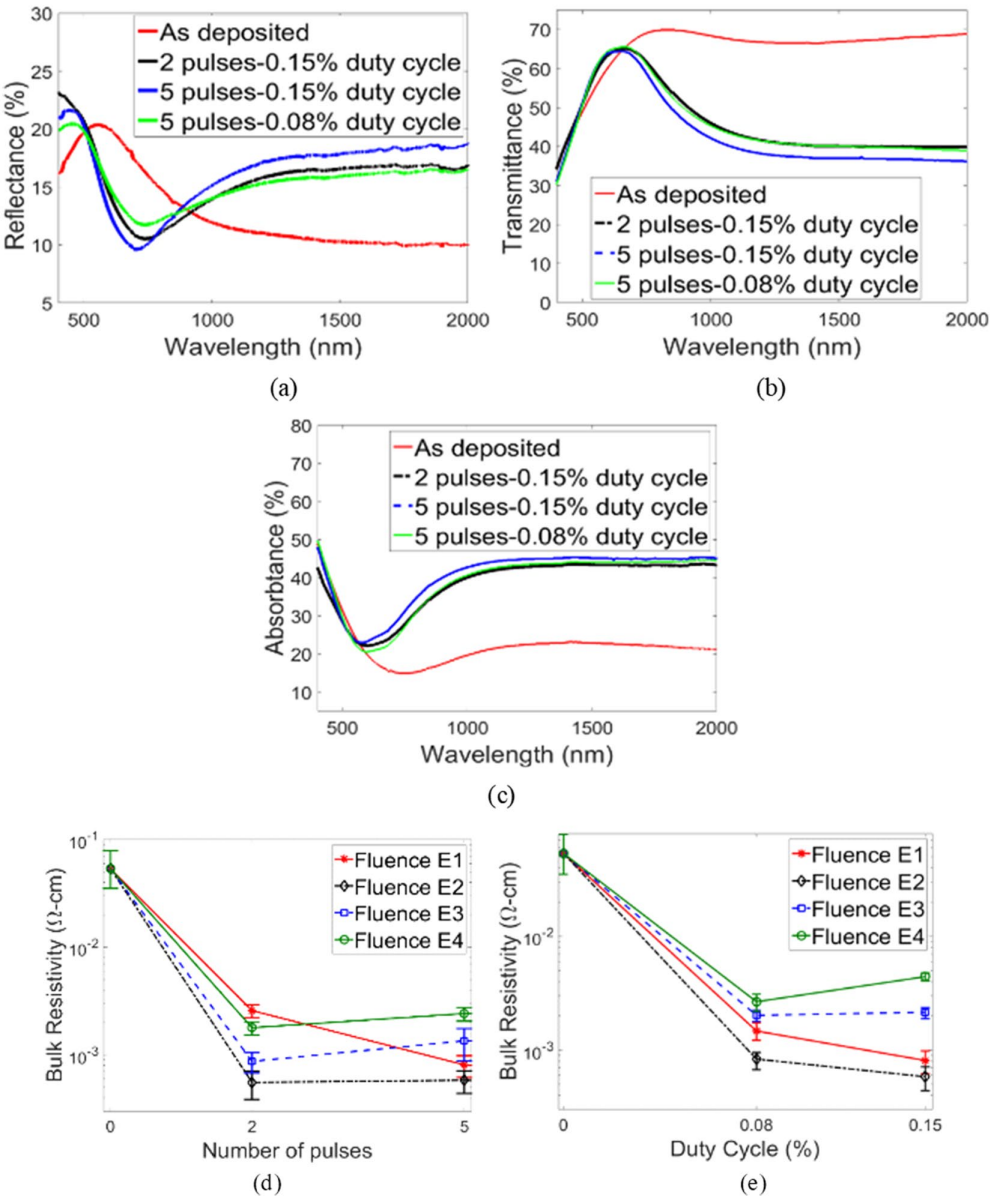
Representative film (**a**) Reflectance (**b**) Transmittance (**c**) Absorbtance at fluence E1. Bulk resistivity for various (**d**) number of pulses (**e**) duty cycle. Markers show average resistivity and error bars show standard deviation in resistivity (calculation method described in Methods section). Zero pulses and duty cycle represent the unsintered film.

Figure 6a–c compare percentage reflectance, transmittance and absorbtance of the post-IPL film for fluence E1 to that of the as-deposited film. Comparisons for additional fluences E2 to E4 are shown in Supplementary Fig. S3a,–c. These optical properties are compared, for different IPL parameters used, at 550 nm (the photopic wavelength for human vision^16^) and at the near-infrared wavelength of 2000 nm in Supplementary Fig. S3d and e respectively. The change in optical properties after IPL at the photopic wavelength is small, e.g., Post-IPL transmittance at 550 nm is 55–65% and that of the as-deposited film is 55% (Fig. S3d). The post-IPL films show a reduction in transmission at 2000 nm by nearly 30%, primarily due to increased absorbtance (Fig. S3e). At a given duty cycle and number of pulses slightly increased transmission and reduced absorption at 2000 nm is observed at higher fluence (e.g. at E4 = 15 J/cm^2^ in Fig. S3e), but without reaching similar levels as the unsintered film. A commonly observed feature of annealing Cu_x_S thin films is a reduction in NIR transmission and increase in NIR absorption, when reduction in bulk resistivity is achieved, as compared to the as-deposited film. This is ascribed to the free-carrier absorption mechanism^16,38^. Figure 6 and Supplementary Fig. S3 show trends in resistivity, NIR transmittance and NIR absorbtance that agree with this past work. The above observed dependence of optical properties on the IPL parameters is explained by the fact that the crystalline phase gradually changes from CuS to Cu_1.8_S with increasing IPL pulse fluence (Fig. 5a–d) and that the Cu_1.8_S phase typically shows lesser absorption and higher transmission in the near-infrared as compared to CuS phase^16,23,39^. The change in near infrared absorption after IPL might also be attributed to thickness reduction but the lack of a trend in the dependence of thickness on fluence does not allow this effect to be easily deconvoluted. These optical properties also show that the use of a thermocouple embedded into the film for measuring temperature evolution^25,26^, would be difficult since the non-negligible visible transmission (e.g., Fig. 6b) would cause significant direct exposure and heating of the thermocouple by the xenon lamp light itself.

Figure 6d,e show that the bulk resistivity of the post-IPL films is around 10^−3^–10^−4^ Ω-cm and is lower than that of the as-deposited film by at least an order of magnitude. Note that the covellite phase of copper sulfide typically shows metal-like conductivity^23^ while the digenite phase exhibits more semiconducting behavior^23,24,39^. For fluence E1, the resistivity monotonically reduces with increasing duty cycle and fluence as the covellite-rich phase develops (Fig. 5a). At fluence E2, the reduction in resistivity with pulse number and duty cycle tapers off as the film develops increasingly similar content of digenite and covellite phases (Fig. 5b). For fluence E3 and E4, the trends are reversed, i.e., increasing duty cycle and pulses result in greater resistivity due to the dominance of the semiconductor-like digenite phase in the film (Fig. 5c,d). Hall effect measurements of the post-IPL films showed greater charge carrier concentration and reduced carrier mobility as compared to the as-deposited film (see Supplementary Fig. S4), and that the variations with IPL parameters are reflective of the corresponding changes in bulk resistivity. As indicated by the positive sign of the Hall coefficient post-IPL films were *p*-type semiconductors, as compared to an indeterminate sign and conductivity type measured for the as-deposited film.

### Theoretical

The absence of an observable temperature turning point in experiments (Fig. 2) and the optical behavior in the 400–700 nm range (Fig. 6a–c and Supplementary Fig. S3) indicates that there is little or no coupling between phase change, NP densification and optical absorption. So the conventional thermal equation for temperature prediction^6,26^ is used and extended here. During IPL on glass substrates, the heat source for the film is the portion of the xenon lamp power that is absorbed by the film, denoted here by *W*. The *W* used in the thermal model was obtained as in equation (1). Here *F* is the cumulative power over the xenon lamp spectrum that is output from the lamp (equation (2)), *P* is the constant power input into the lamp, *λ* is the wavelength, *X*(*λ*) is the fractional power spectrum of the xenon lamp as supplied by the manufacturer (see Supplementary Fig. S5) and *A*(*λ*) is the fractional absorption by the Cu_x_S film.

1 $$W=\frac{F\sum _{\lambda =400\,nm}^{\lambda =700\,nm}A(\lambda )\cdot X(\lambda )}{\sum _{\lambda =400\,nm}^{\lambda =700\,nm}X(\lambda )}$$

2 $$F=P\sum _{\lambda =400\,nm}^{\lambda =700\,nm}X(\lambda )$$

**Figure 7:**
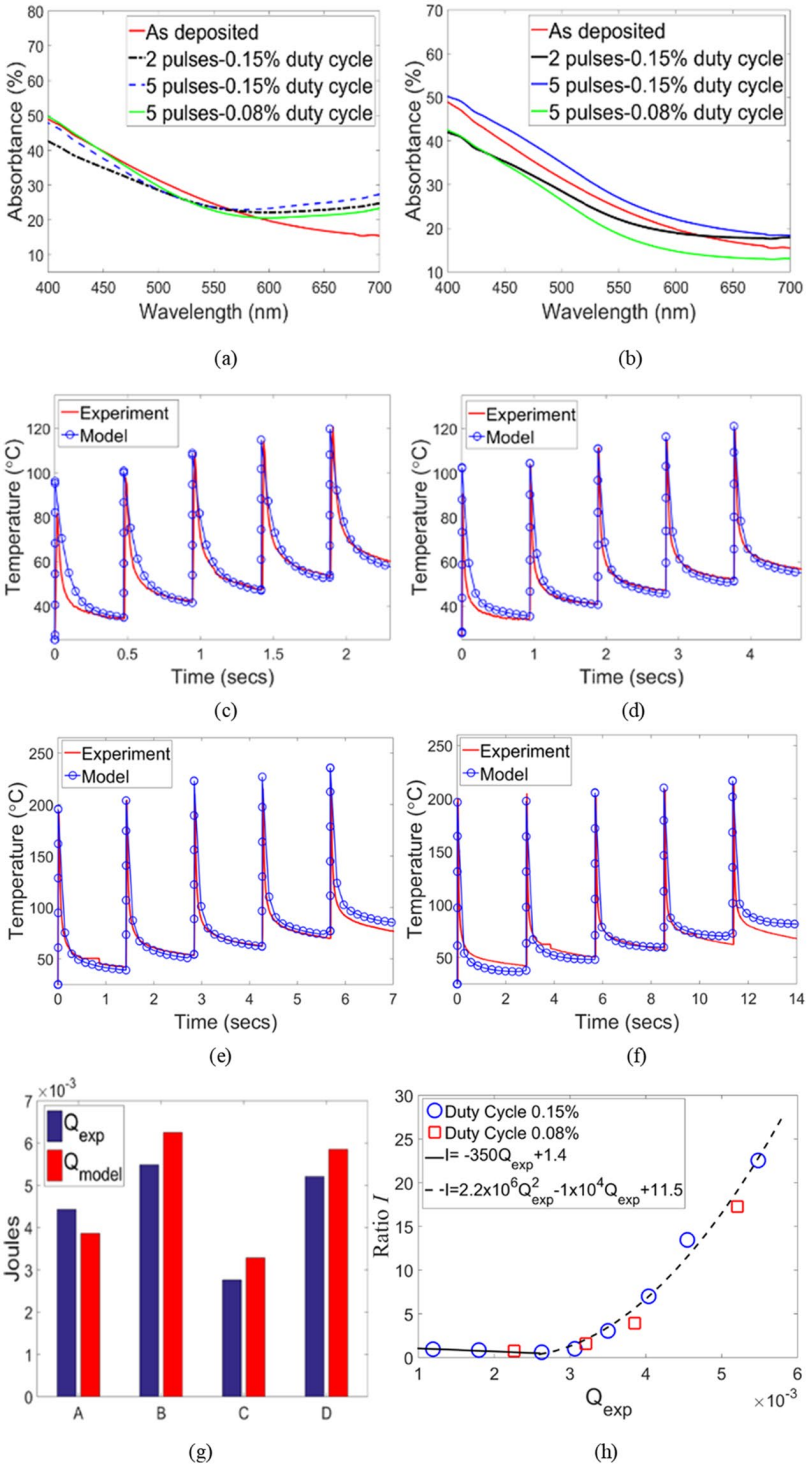
Optical absorption of as-deposited and post-IPL films with fluence (**a**) E1 (**b**) E4. Predicted and experimental film temperature for (**c**) fluence E1-5 pulses-0.15% duty cycle (**d**) fluence E1-5 pulses-0.08% duty cycle (**e**) fluence E4-5 pulses-0.15% duty cycle (**f**) fluence E4-5 pulses-0.08% duty cycle. (**g**) Experimental and predicted dissipated energy in the film. A: fluence E1-5 pulses-0.15% duty cycle, B: fluence E1-5 pulses-0.08% duty cycle, C: fluence E4-5 pulses-0.15% duty cycle, D: fluence E4-5 pulses-0.08% duty cycle (**h**) I vs Q_exp_ plot.

This form of *W* accounts for the absorption characteristics of the Cu_x_S thin film. The summation in equation (1) was performed from *λ* = 400 to 700 nm, since most of the energy of the xenon lamp light is concentrated in this wavelength range. The optical energy of the lamp in the near-infrared range is no more than 5% of the total lamp energy and any wavelengths below than 300 nm are filtered out by the xenon lamp’s window. The optical absorbtance of the film within this range (Fig. 7a,b and Supplementary Fig. S6) showed that the difference in cumulative visible optical absorbtance between as-deposited and post-IPL films is no more than 10%. Thus, the function *A(λ)* was fixed as that obtained from the absorption curves of the as-deposited film. Given the small film thickness, the xenon lamp light was assumed to fully penetrate the film. Since the nanometer scale film thickness also implies a small Biot number and thus a negligible conductivity induced thermal gradient within the film^27^, *W* was assumed to be uniformly distributed in the bulk of the film. Note that the negligible change in visible optical absorbtance as a function of IPL parameters implies insignificant change in the magnitude of *W* with densification and phase change during IPL. The coupling between densification and optical absorption in metal NPs is due to their nanoshape-dependent plasmonic behavior in the visible spectrum, so that as NP fusion changes the nanoscale shape the degree of plasmonic behavior changes as well^3^. Cu_x_S does not show plasmonic behavior in the visible spectrum and its polymorphs show negligible differences in visible absorbtance, so that even with phase change and NP fusion there is little change in xenon light absorption. This explains the lack of a self-limiting behavior and temperature turning point in IPL of Cu_x_S NP films.

Figure 7c–f show that there is good agreement between theoretically predicted and experimentally measured film temperatures for the validation cases examined here. The maximum error in peak pulse temperature prediction is 20 °C and occurs in the first pulse with the lowest fluence E1.

As is well known from past work on conventional annealing of the Cu_x_S system^12,18,34,40^ the phase change, for a given starting composition of the as-deposited film, is dictated by the temperature history of the film, i.e., a combination of temperature and time. The complex temperature history of the film in IPL can be captured via as the thermal energy dissipated into the film due to IPL. The experimentally observed dissipated energy *Q*_*exp*_ was calculated using equation (3), where *T*_*exp*_ is the experimentally measured film temperature, *ρ* and *C*_*p*_ are the density and specific heat capacity of the film (values shown in Supplementary Table S1), *t*_*film*_ is the film thickness fixed at 115 nm (i.e., average as-deposited film thickness in Fig. 4a,b), *A*_*film*_ is the in-plane area of the as-deposited film, and *t* is time. The theoretically predicted dissipated energy after IPL, i.e., *Q*_*model*_, was obtained directly from COMSOL. Figure 7g shows that the thermal model yields good agreement between *Q*_*exp*_ and *Q*_*model*_ (maximum error 15%).

3 $${Q}_{\exp }=\rho {t}_{film}{A}_{film}{C}_{p}{\int }_{t=0}^{t={\rm{end}}\,{\rm{of}}\,{\rm{IPL}}}{\dot{T}}_{\exp }dt$$

The experimentally observed change in relative degree of digenite to covellite phase content in the film was quantitatively captured as a scalar ratio *I*, defined as the ratio of intensity of the 2θ = 46.42° major Cu_1.8_S peak to that of the 2θ = 48.06° major CuS peak in GIXRD data (Fig. 5a–d). When *I* is greater than 1 then the digenite phase dominates, and the greater the value of *I* the greater the digenite content relative to the covellite phase. When *I* is lesser than 1 then the covellite phase dominates, and the smaller the value of *I* the greater the covellite content relative to the digenite phase. If we only consider the experiments where the pulse fluence and pulse number are varying at constant duty cycle of 0.15% (blue circles in Fig. 7h), a relationship between *I* and *Q*_*exp*_ emerges that can be approximated as a linear function when *Q*_*exp*_ ≤ 2.6 millijoules and as a quadratic function when *Q*_*exp*_ > 2.6 millijoules. The cases with 0.08% duty cycle (red squares in Fig. 7h) follow this relationship as well, validating its applicability.

Figure 7h also shows that an increasingly covellite phase is formed as *Q*_*exp*_ approaches 2.6 millijoules, i.e., *I* < 1 and *I* is reducing in magnitude with increasing *Q*_*exp*_. When *Q*_*exp*_ is greater 2.9 millijoules then *I* > 1 and its value increases with increasing *Q*_*exp*_, i.e., an increasingly digenite rich phase is formed with increasing *Q*_*exp*_. When *Q*_*exp*_ is between 2.6 to 2.9 millijoules the dominant phase is still covellite (i.e., *I* is still lesser than 1) but the value of *I* starts to increase and tends towards 1 with increasing *Q*_*exp*_, indicating that this is an intermediate region where the covellite phase is still dominant but the dominance of the digenite phase is incipient. This observation agrees with past work which shows that the heat of formation of covellite is lower than that of digenite^41^.

**Figure 8:**
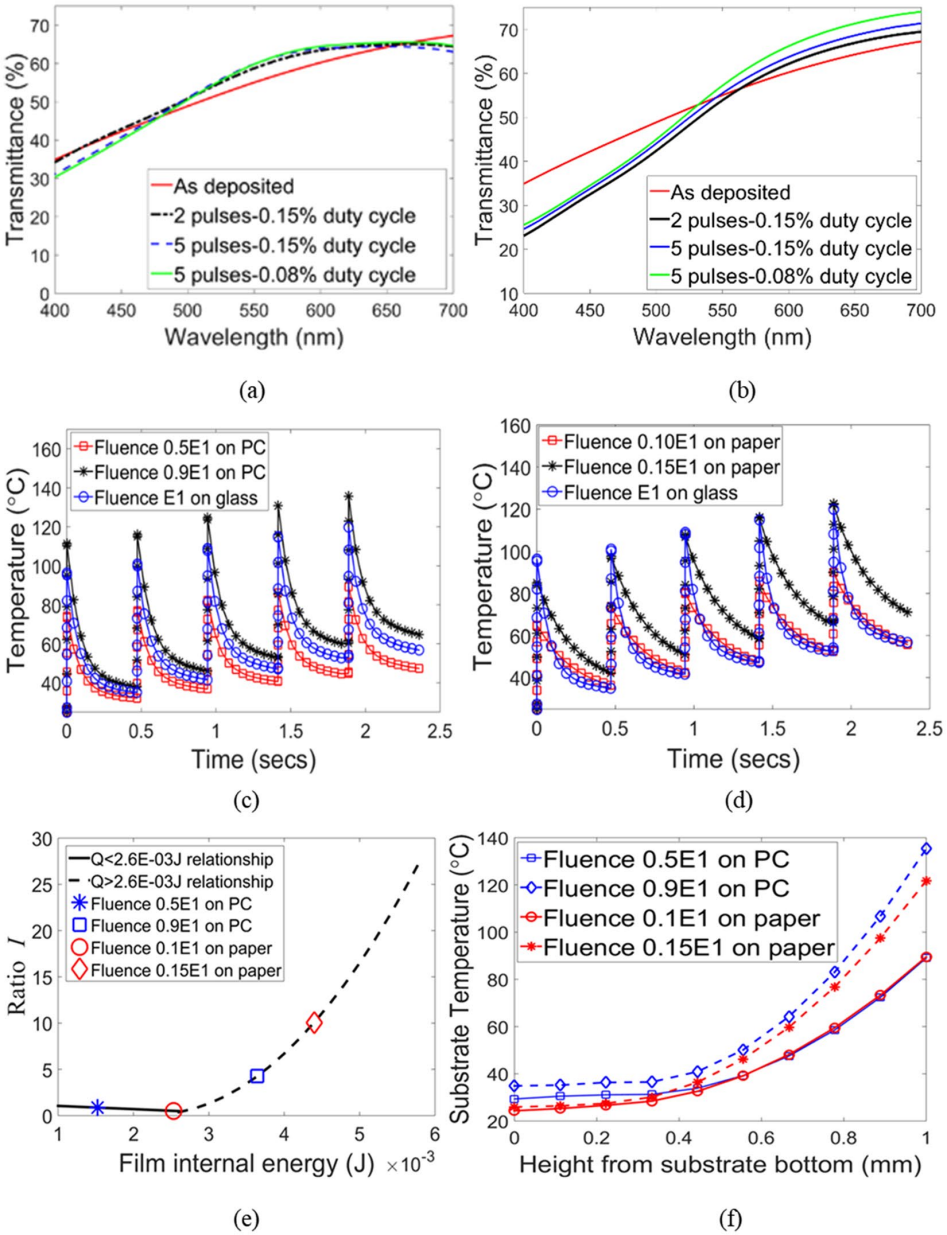
Film transmittance in 400–700 nm range for fluence (**a**) E1 (**b**) E4. Predicted film temperature for (**c**) PC and (**d**) paper substrates. (**e**) Predicted film phase for PC and paper substrates. (**f**) Predicted substrate temperatures at end of last pulse for PC and paper substrates. 0 indicates bottom of substrate. All cases shown for 0.15% duty cycle.

Since there is good agreement between predicted and experimental dissipated energy (Fig. 7g) the *Q*_*exp*_ on the x-axis in Fig. 7h can be replaced with *Q*_*model*_, which enables us to use the validated thermal model (Fig. 7c–f) to predict post-IPL phase when substrates besides glass are used. To do so, the glass substrate in the thermal model was replaced with polycarbonate (PC) and paper of the same thickness as the glass, along with the appropriate thermal properties of PC and paper (see Supplementary Table S1). The PC was assumed to be visibly transparent, so that the xenon light transmitted through the Cu_x_S film did not heat up the PC directly. The paper substrate was assumed to be visibly opaque, due to which the portion of the xenon lamp light transmitted through the film would directly heat up the surface of the paper at the film-paper interface. To capture this phenomenon a boundary heat source *Q*_*b*_ was specified at the film-paper interface (Fig. 2e) using the wavelength dependent transmission spectrum of the Cu_x_S film *Tr(λ)*, as shown in equation (4). Since there was negligible change in the cumulative film transmittance within the 400–700 nm wavelength range after IPL (Fig. 8a,b and Supplementary Fig. S7) the function *Tr(λ)* was fixed as that obtained from the as-deposited film.

4 $${Q}_{b}=\frac{F\sum _{\lambda =400\,nm}^{\lambda =700\,nm}Tr(\lambda )\cdot X(\lambda )}{\sum _{\lambda =400\,nm}^{\lambda =700\,nm}X(\lambda )}$$

Using the energy dissipated in the film obtained from these thermal simulations, and the *I* versus dissipated energy relationship shown in Fig. 7h, the phase of the film was predicted for the cases of paper and PC substrate being used. No extrapolation was performed or needed beyond the range of *Q*_*exp*_ shown in Fig. 7h. For the PC substrate at fluence 0.5 × E1 the peak film temperature is lower than 100 °C (Fig. 8c) and a primarily covellite phase is formed (*I* = 0.87, Fig. 8e). Fluence 0.9 × E1 causes higher maximum film temperature than with fluence E1 on the glass substrate and the corresponding film phase after IPL is primarily digenite (*I* = 4.261, Fig. 8e). The higher peak temperature per unit fluence as compared to glass substrates is primarily due to lower thermal conductivity of PC as compared to that of glass. For the paper substrate, the peak film temperature per unit fluence is significantly higher than for glass and PC substrates (Fig. 8d). Low fluences of 0.10 × E1 and 0.15 × E1 are sufficient to raise the film temperature and dissipated energy enough to create covellite (*I* = 0.52, Fig. 8e) and digenite rich phases (*I* = 10.1, Fig. 8e) respectively. This is due to the lower thermal conductivity of paper (0.05 W/m-K) as compared to PC and glass, and due to the presence of a boundary heat source at the substrate-film interface due to the visible opacity of paper.

Further, Fig. 8e shows that a more strongly covellite-rich phase (lesser *I* while *I* < 1) is formed with much lesser fluence for the paper substrate than with the PC substrate. The same is true for the formation of the digenite phase with paper substrate as compared to the PC substrate. Note that using the same fluence for PC and paper substrates as for the glass substrate will result in greater temperatures than those predicted in Fig. 8c,d, formation of only digenite-rich films, and potentially greater film oxidation. These observations indicate that the control of IPL parameters to achieve a desired film phase must carefully consider the optical (both transmittance and absorbtance) and the thermal properties of the film and the substrate, rather than just the optical absorbtance of the film and the thermal properties of the film and the substrate.

Figure 8f compares thermal penetration into the PC and paper substrates at the end of the last pulse on-time, i.e., when the film and substrate temperature are highest. While the top surface of the substrate is at the film temperature the temperature drops by 65–70% at a depth of about 50% into the substrate. The peak temperatures experienced by the substrates are lower than the glass transition temperature for PC (150 °C) and the ignition temperature for paper (233 °C), indicating the usability of these materials as substrates for IPL of Cu_x_S thin films. Thus, the extended model developed here enables a-priori control of IPL parameters to achieve desired film phase, and concurrently allows assessment of substrate temperature gradients that may cause substrate damage and distortion.

## Discussion

This work demonstrates and characterizes IPL of Cu_x_S NP thin films, and predicts the temperature evolution and crystal phase change in the film. The as-deposited film with Cu to S stoichiometry of 1.8 loses sulfur due to evaporation during IPL, crystallizing to a covellite-rich and then to a digenite-rich phase as the maximum film temperatures during IPL increase. This phase evolution agrees with the copper-sulfide phase diagram^37^ and is qualitatively consistent with the energies of formation required for these phases^41^. This phase change is accompanied by a reduction in film thickness and roughness due to NP fusion and sulfur evaporation. Post-IPL films show little change in visible transmission at the photopic wavelength (i.e., 550 nm). However, infrared transmission of the post-IPL films is significantly lower than that of the as-deposited film, with a slight increase as a more digenite-rich phase is formed. This optical behavior is likely due to a combination of the change in crystal phase^17,18,23^ and the change in film morphology and thickness. The post-IPL films have p-type conductivity and the changes in bulk resistivity, carrier concentration and carrier mobility as a function of crystal phase are similar to past work on annealing of Cu_x_S films^10,24^.

Experimentally measured temperatures show no self-limiting phenomenon in IPL of Cu_x_S NP films, unlike IPL of metal NPs^3^, because the change in optical absorption (within 400–700 nm) with change in phase or densification is negligible. The temperatures at which a crystalline covellite phase is obtained from amorphous as-deposited films in IPL is around 126 °C and recrystallization temperature to digenite is around 155 °C, i.e., 37–39% lower than the annealing temperatures used in past work^35^. The time scales in which the recrystallization is obtained is seconds as compared to hours in conventional thermal annealing^18,24,42^ or minutes in Rapid Thermal Annealing^39^. This is likely due to a combination of the high specific surface energy of NPs^36^, and rapid localized heating of the film by the xenon lamp light. Also, the large-area of the xenon lamp (≥12 × 0.75 sq. inches here) shows the potential for scalable fabrication.

The thermal model implemented here predicts film temperature evolution that agrees with experimental measurements, and the phase content and dissipated energy in the film are shown to be closely correlated. The use of dissipated energy in the film, rather than the film temperature, to predict phase evolution allows the combination of temperature and time to be accounted for. This correlation between dissipated energy and change in phase of the film is qualitatively similar to the energy of formation of one polymorph of Cu_x_S from the other. Thus, this model would be usable whether fluence is being changed via only changing lamp voltage, or lamp on-time, or both simultaneously. By combining this thermal model with the thermodynamic approach for phase prediction we show that covellite and digenite phases can be formed with lesser fluence on paper and PC substrates, than with glass substrates. Thus, obtaining a desired phase composition in the Cu_x_S film (or any such NP film that changes crystal phase and has some transmittivity in the visible spectrum) requires control of IPL parameters to account for thermal and optical properties of the substrate, and thermal properties and visible optical transmissivity (and not just absorptivity) of the film. The extended model developed in this work can enable this type of a-priori process control. Future work by the authors will focus on combining the above modeling approach with *in situ* measurement of film properties, based on past work^25^, and testing of the mechanical and environmental durability of IPL processed Cu_x_S thin films.

## Methods

The Sinteron 3000 Xenon lamp had an optical footprint of 12 inches by 0.75 inch at 1 inch distance from the lamp. The samples were mounted on a stationary platform such that the entire film surface was within the optical footprint of the lamp. The film emissivity was manually calibrated by heating the film to a known temperature of 80 °C on a hot plate within the above described IPL setup. The lamp’s operating voltage *V* and pulse on-time *t*_*on*_ determine the incident pulse fluence *E*_*p*_ as per the relationship *E*_*p*_ = *t*_*on*_ × (*V*/3120)^2.4^ supplied by the lamp manufacturer. For all experiments performed here the voltage *V* was kept constant at 3000 V and the increase in *E*_*p*_ was effected by increasing *t*_*on*_. The minimum off-time (governed by the charging time for the discharge capacitors) was used as the off-time for all cases with 0.15% duty cycle, and the off-time was increased to enable duty cycle of 0.08%. Changes in film morphology was characterized using cross-sectional Scanning Electron Microscopy (SEM) with a FEI Quanta 3D dual beam system. These scans were also used to obtain the film thickness over at least 10 different locations for each IPL parameter combination. A Bruker Innova Atomic Force Microscope (AFM) was used in tapping mode to determine the surface roughness (arithmetical mean deviation R_a_) of the films. The elemental film composition was characterized via a FEI QUANTA 600 F SEM X-Ray Energy Dispersive Spectrometer (EDS). At least five measurements were taken at different film locations for each IPL parameter combination. The films’ crystallinity and phase was identified using a Rigaku Ultima-IV X-ray diffractometer in Glancing Incidence mode from 2θ = 10° to 60° at a resolution of 0.02° with a fixed grazing angle of 0.35°. The percentage transmittance *T* and reflectance *R* were measured within a spectral range of 300 nm to 2000 nm with a resolution of 0.2 nm using a JASCO V670 UV-Visible-NIR spectrophotometer equipped with an integrating sphere. The absorbtance *A* was obtained as *A* = 1 − *T* − *R*. Sheet resistance was measured using a Signatone four-point probe over at least 10 sampling points. The average bulk resistivity was calculated using the average thickness and the average sheet resistance and the standard deviation in bulk resistivity was obtained using the average thickness and the measured standard deviation in sheet resistance. Charge carrier concentration and mobility were quantified via Hall Effect measurements (Ecopia HMS-5000) in the Van der Pauw configuration at room temperature, at a constant current of 5 mA, and in a magnetic field of 0.5 Tesla. At least five measurements were made for each combination of IPL parameters used.

The Finite element model (supplementary Fig. S8) consisted of a film of the same thickness as in experiments, and two 10 nm thick layers of substrate material below it. The lowermost layer of substrate was modeled as an infinite element domain with a pole distance equal to the thickness of the substrate. This allowed modeling of the full thickness of the substrate as used in experiments. The meshing was performed so that the elements in both the film and the substrate were smaller near the film-substrate interface and larger farther away from it. The in-plane mesh size was 1 nm. Since symmetry boundary conditions were used on the side-walls of the model (as in Fig. 1e), the in-plane size of the model was fixed at 10 nm to keep computational time low.

### Data Availability

The datasets generated during and/or analysed during the current study are available from the corresponding author on reasonable request.

## Electronic supplementary material

Supplementary information
