# Author Correction: Temperature, Crystalline Phase and Influence of Substrate Properties in Intense Pulsed Light Sintering of Copper Sulfide Nanoparticle Thin Films

**DOI:** 10.1038/s41598-020-60912-8

**Published:** 2020-02-28

**Authors:** Michael Dexter, Zhongwei Gao, Shalu Bansal, Chih-Hung Chang, Rajiv Malhotra

**Affiliations:** 1 Department of Mechanical and Aerospace Engineering, Rutgers University, Piscataway, New Jersey 08854 USA; 2 Department of Chemical Engineering, Oregon State University, Corvallis, Oregon 97331 USA; 3 School of Mechanical, Industrial and Manufacturing Engineering, Oregon State University, Corvallis, Oregon 97331 USA

Correction to: *Scientific Reports* 10.1038/s41598-018-20621-9, published online 02 February 2018

This Article contains errors in Reference 9 which was incorrectly given as:

Williams, B. A., Smeaton, M. A., Holgate, C. S., Francis, L. F. & Aydil, E. S. Effect of intense pulsed light annealing on the microstructure of copper zinc tin sulfide nanocrystal coatings. *Journal of Vacuum Science & Technology A: Vacuum, Surfaces, and Films* **34**, 1–28, doi:10.1116/1.4961661 (2015).

The correct reference is listed below as ref. 1
