# Supplementary material for: Temperature, Crystalline Phase and Influence of Substrate Properties in Intense Pulsed Light Sintering of Copper Sulfide Nanoparticle Thin Films
Source: Sci Rep. 2018 Feb 2;8:2201. doi: 10.1038/s41598-018-20621-9 (PMC5797230; doi:10.1038/s41598-018-20621-9)
Supplement: Supplementary file 1 — Supplementary information [file 41598_2018_20621_MOESM1_ESM.pdf]

Temperature, Crystalline Phase and Influence of Substrate Properties in Intense Pulsed Light Sintering of Copper Sulfide Nanoparticle Thin Films

Michael Dexter, Zhongwei Gao, Shalu Bansal, Chih-Hung Chang and Rajiv Malhotra*

Discussion S1: The chemical bath was composed of 7.49 grams of copper (II) sulfate ($CuSO_4 \cdot 5H_2O$ Alfa Aesar), 2.46 grams of sodium acetate (CH_3COONa, Macron), 33.78 grams of triethanolamine ($C_6H_{15}NO_3$, Sigma) and 15.2 ml ammonium hydroxide (28%.0-30.0% NH_4OH, Macron) dissolved in 100 ml of DI water. Another 20 ml of 1.5M thiourea (CH_4N_2S, Sigma-Aldrich) solution was added to the bath, followed by additional DI water. The chemical bath was stirred and placed into a water bath at a temperature of 45 ºC on a hot plate. The glass substrates were immersed vertically into the chemical bath solution for 50 minutes and the film grew on the substrates. After deposition, the substrates were rinsed in DI water and dried using nitrogen gas.

Table S1. Thermal properties and model parameters used in theoretical model. *K* is thermal conductivity, C_p is specific heat capacity and ρ is density.

Cu_xS film[1,2]	K = 2 W/m-K; C_p=450 J/Kg-K; ρ = 5600 kg/m^3
Glass substrate	K = 0.6 W/m-K; C_p=840 J/Kg-K; ρ = 2500 kg/cm^3 (for glass slides)
Polycarbonate substrate	K = 0.19 W/ m-K; C_p = 1250 J/kg-K; ρ = 1200 kg/cm^3 (for optical grade polycarbonate)
Paper substrate	K = 0.05 W/m-K; C_p= 1340 J/Kg-K; ρ = 800 kg/cm^3 (for printing paper)
Convective heat transfer coefficient between Cu_xS film and air	10 W/m^2-K

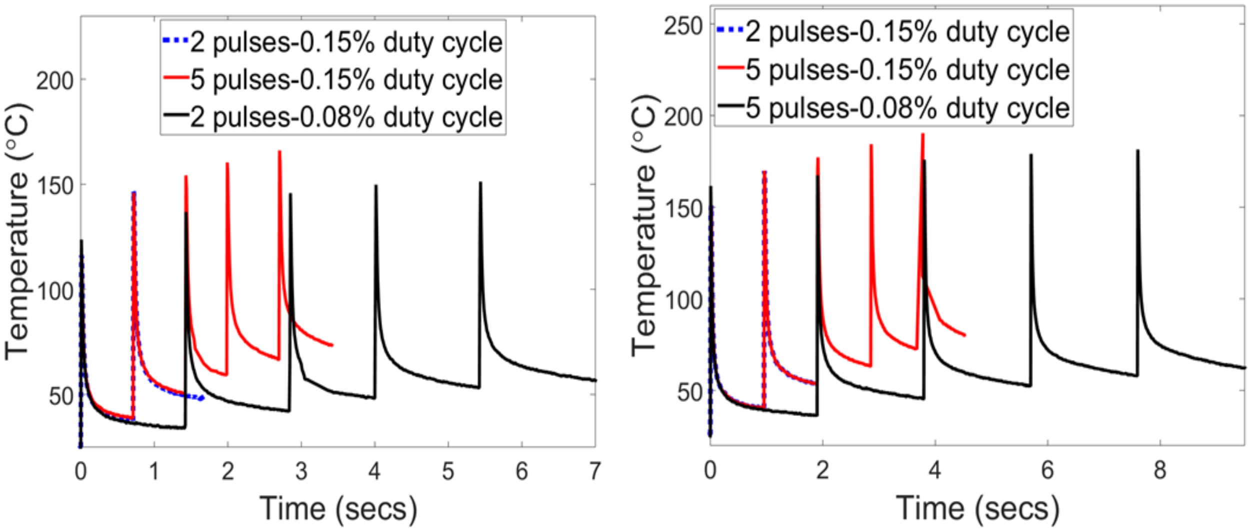

(a) (b)

Figure S1: Temperature evolution plots for different pulse duty cycle and number of pulses for pulse fluence (a) E2 and (b) E3.

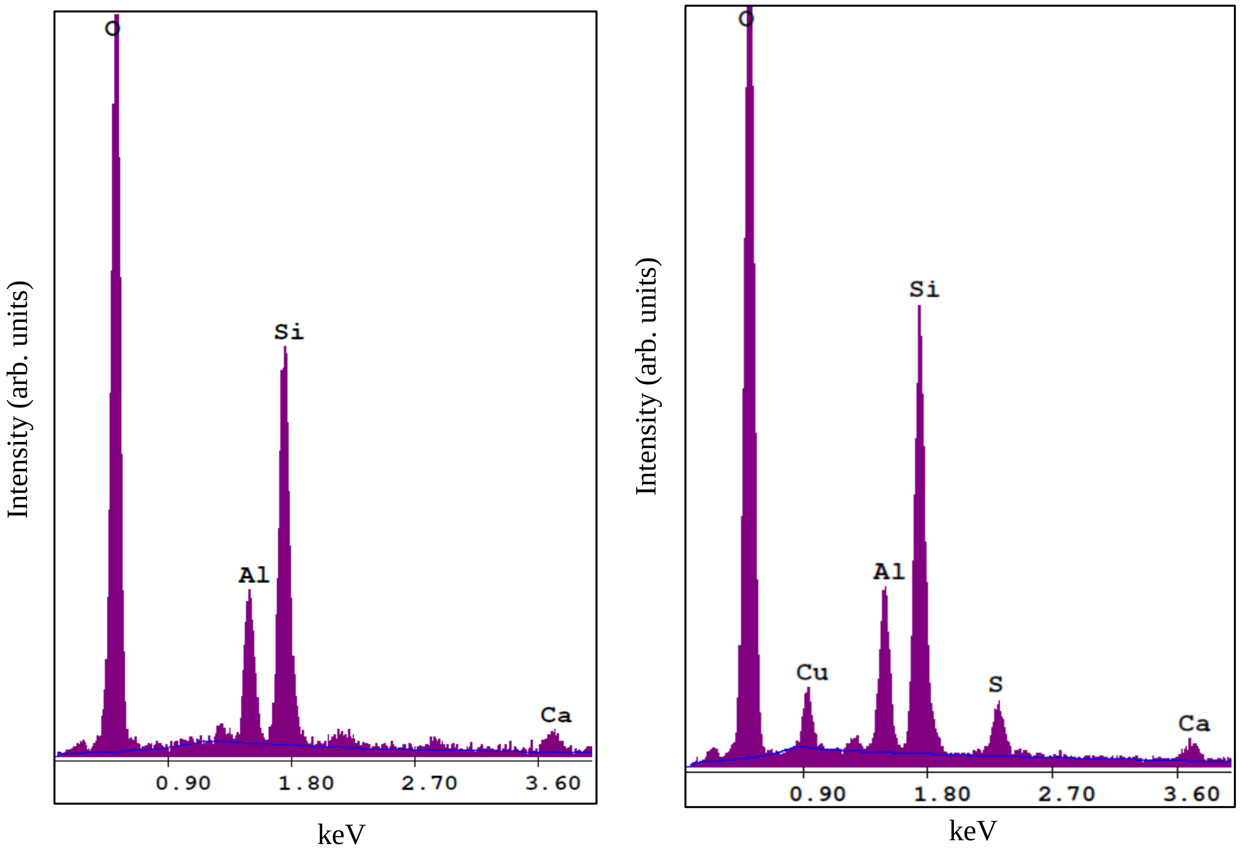

(a) (b)

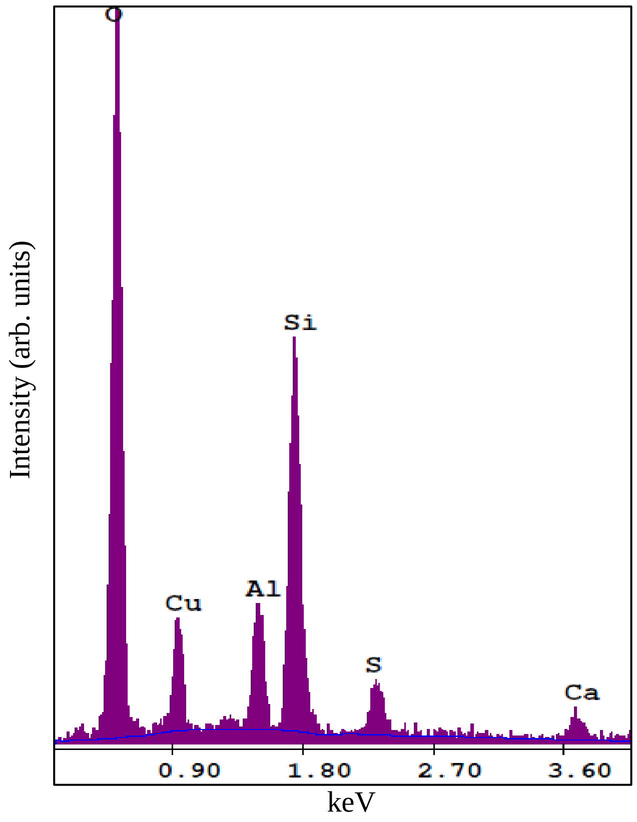

(c)

Figure S2: Representative EDS spectra for (a) glass substrate (b) as-deposited film on glass substrate (c) Post-IPL film sintered with fluence E1-5 pulses-0.15% duty cycle.

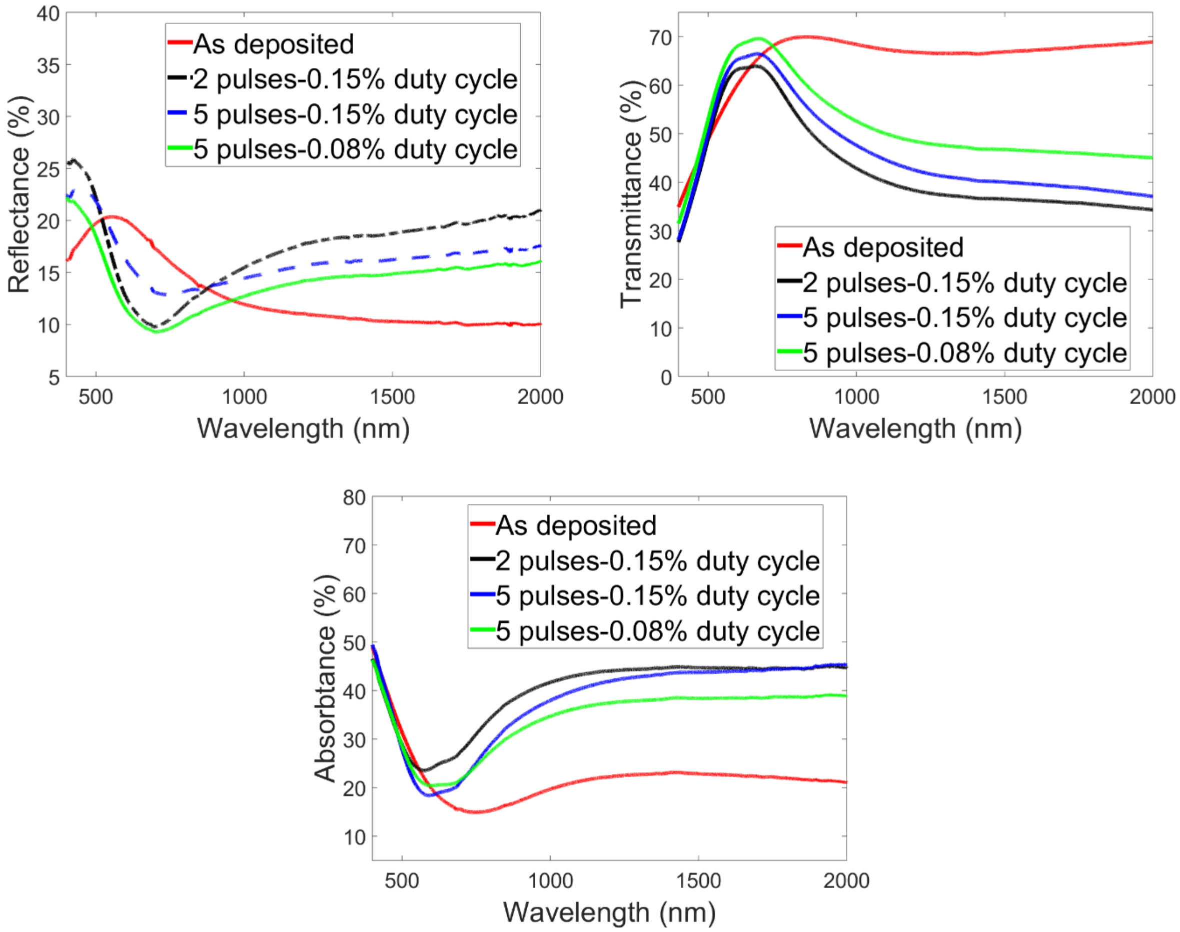
Reflectance (%)
Wavelength (nm)
Transmittance (%)
Absorbtance (%)
As deposited
2 pulses-0.15% duty cycle
5 pulses-0.15% duty cycle
5 pulses-0.08% duty cycle

(a)

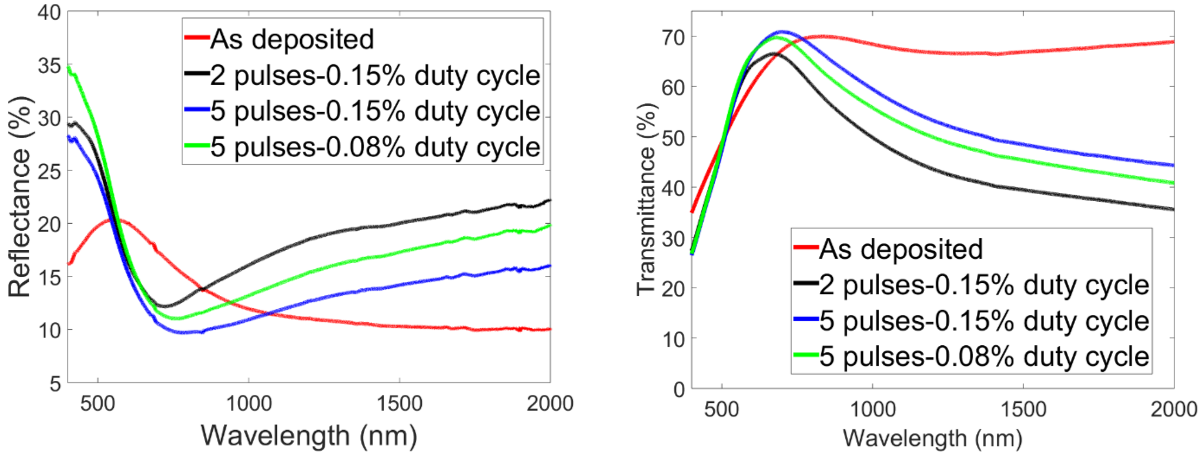
Reflectance (%)
Wavelength (nm)
Transmittance (%)
As deposited
2 pulses-0.15% duty cycle
5 pulses-0.15% duty cycle
5 pulses-0.08% duty cycle

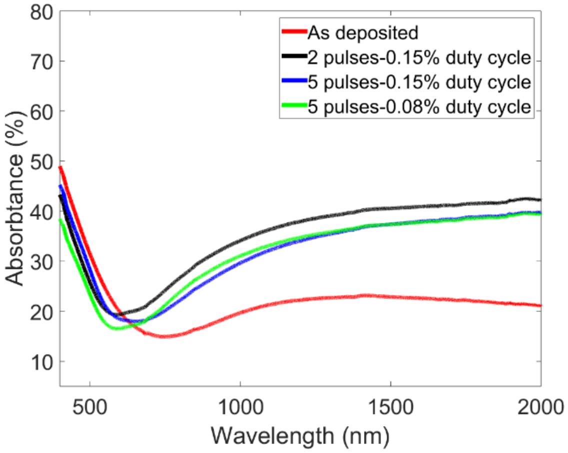
As deposited
2 pulses-0.15% duty cycle
5 pulses-0.15% duty cycle
5 pulses-0.08% duty cycle
Absorbance (%)
Wavelength (nm)
80
70
60
50
40
30
20
10
500
1000
1500
2000

(b)

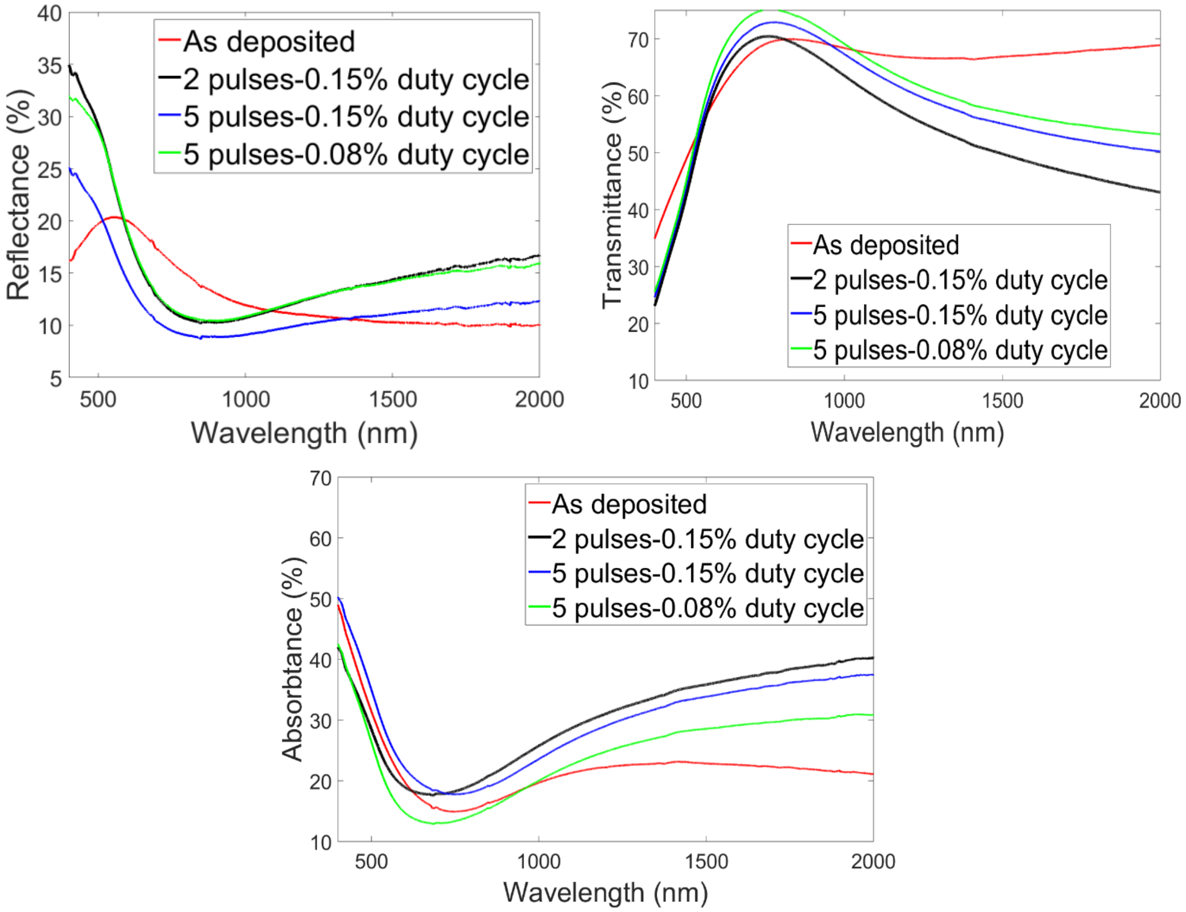
As deposited
2 pulses-0.15% duty cycle
5 pulses-0.15% duty cycle
5 pulses-0.08% duty cycle
Reflectance (%)
Transmittance (%)
Absorbance (%)
Wavelength (nm)
500
1000
1500
2000

(c)

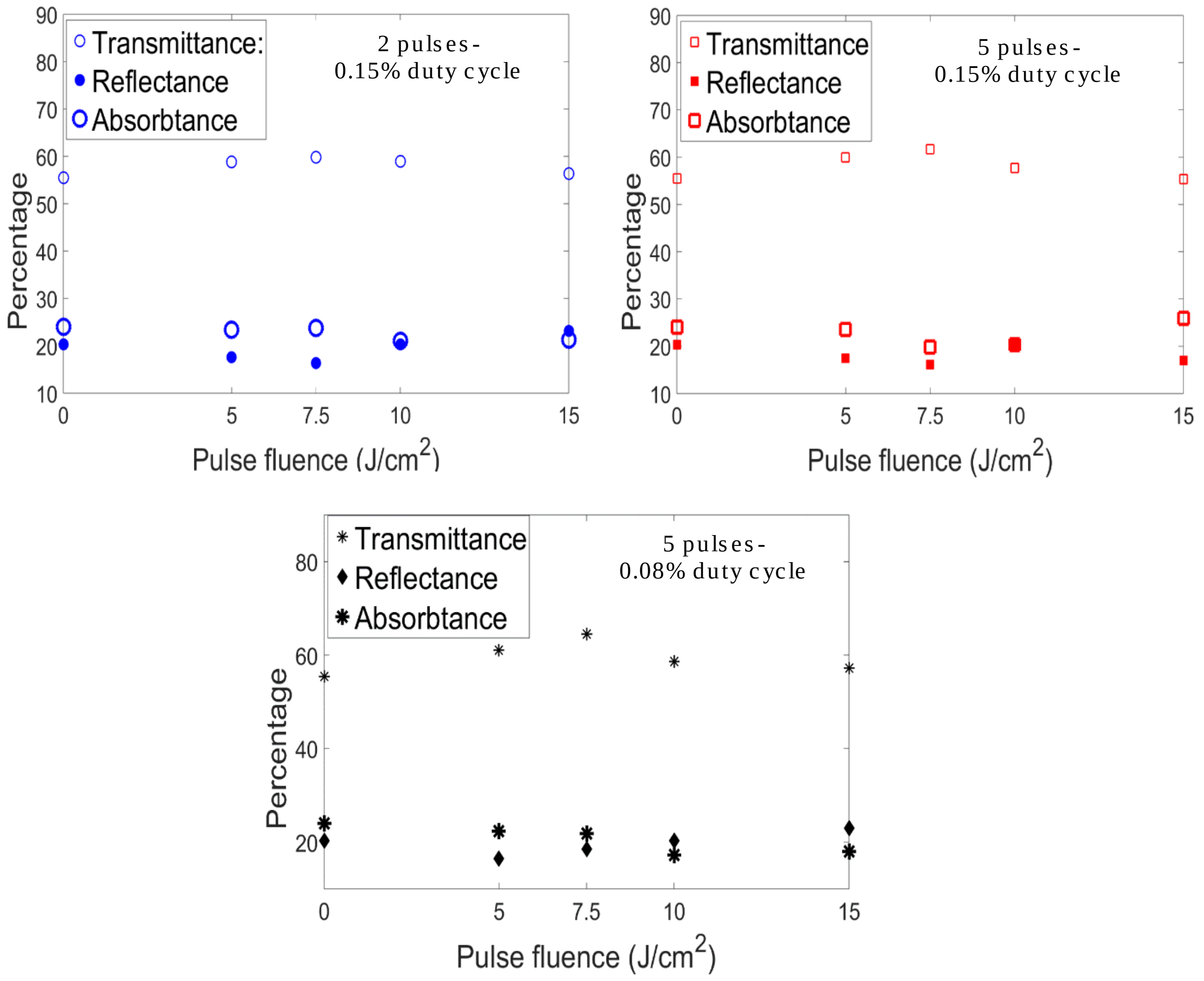
Transmittance:
Reflectance
Absorbtance
2 pulses-
0.15% duty cycle
Percentage
Pulse fluence (J/cm2)
Transmittance
Reflectance
Absorbtance
5 pulses-
0.15% duty cycle
Percentage
Pulse fluence (J/cm2)
Transmittance
Reflectance
Absorbtance
5 pulses-
0.08% duty cycle
Percentage
Pulse fluence (J/cm2)
(d)

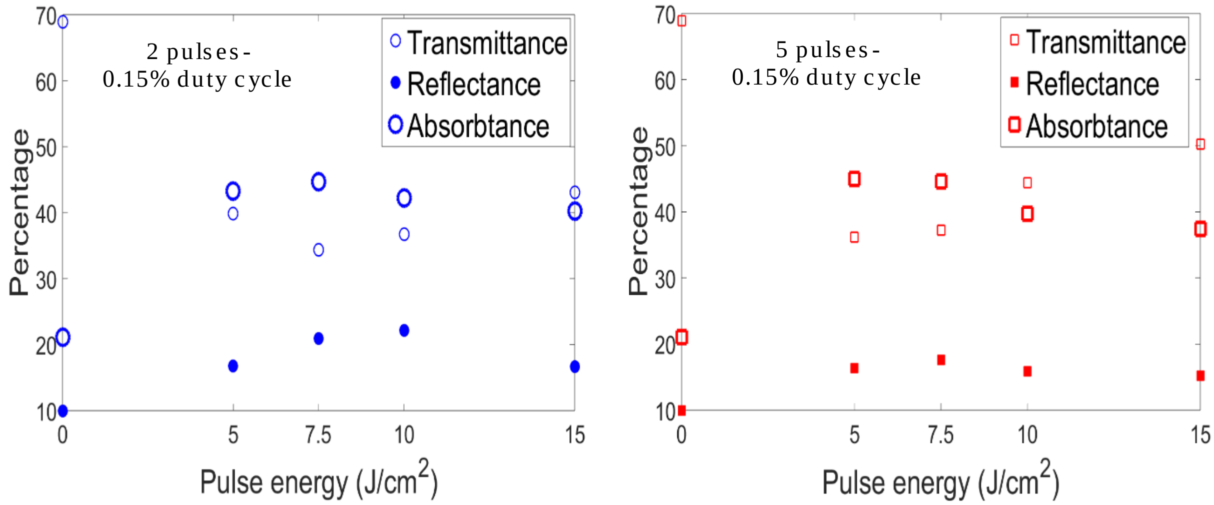

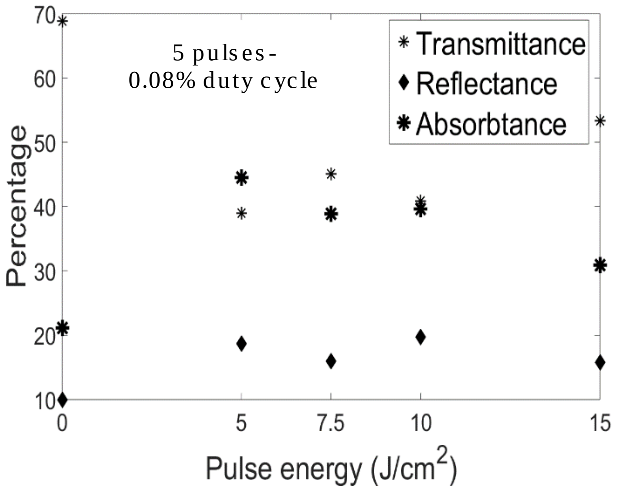

(e)

Figure S3: Reflectance, Transmittance and Absorbtance of as-deposited and post-IPL Cu_xS films for fluence (a) E2 (b) E3 (c) E4. Film transmittance, reflectance and absorbtance at (d) 550 nm (e) 2000 nm.

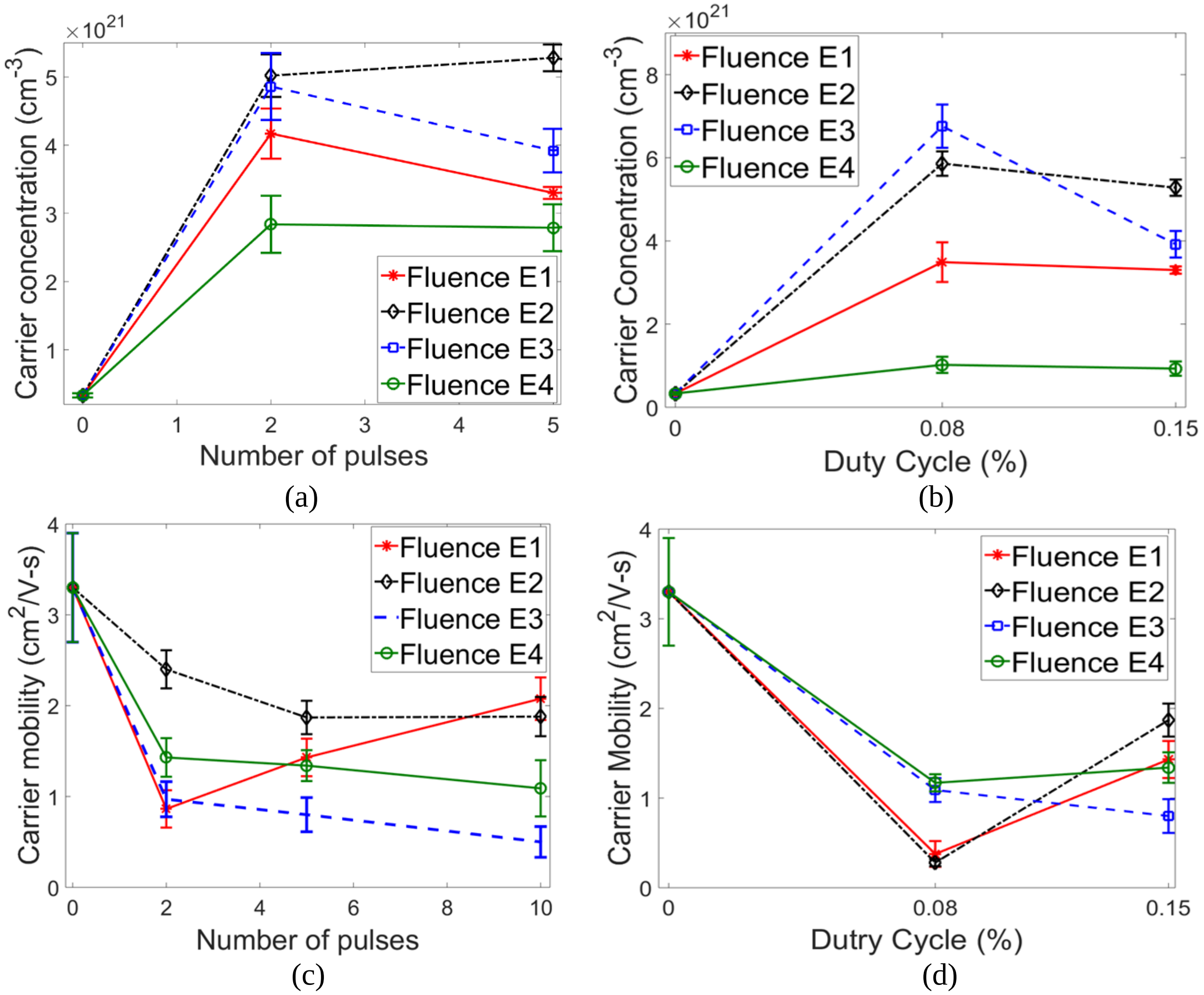

Figure S4: Charge carrier concentration for various (a) number of pulses (b) duty cycle. Charge carrier mobility for various (c) number of pulses (d) duty cycle. Markers show average values and error bars show standard deviation over five measurements. Zero pulses and duty cycle represent the unsintered film.

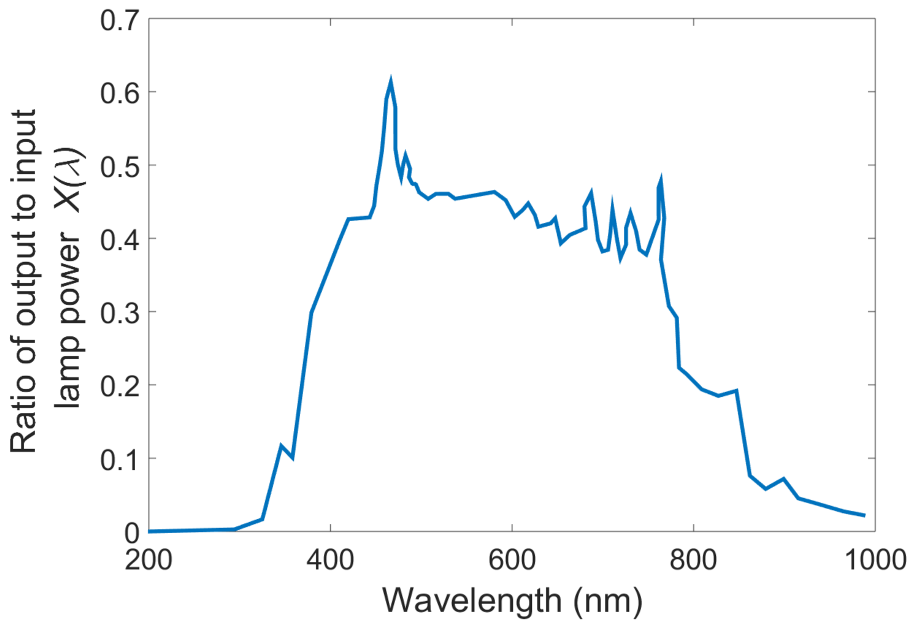

Figure S5: Power spectrum of the xenon lamp.

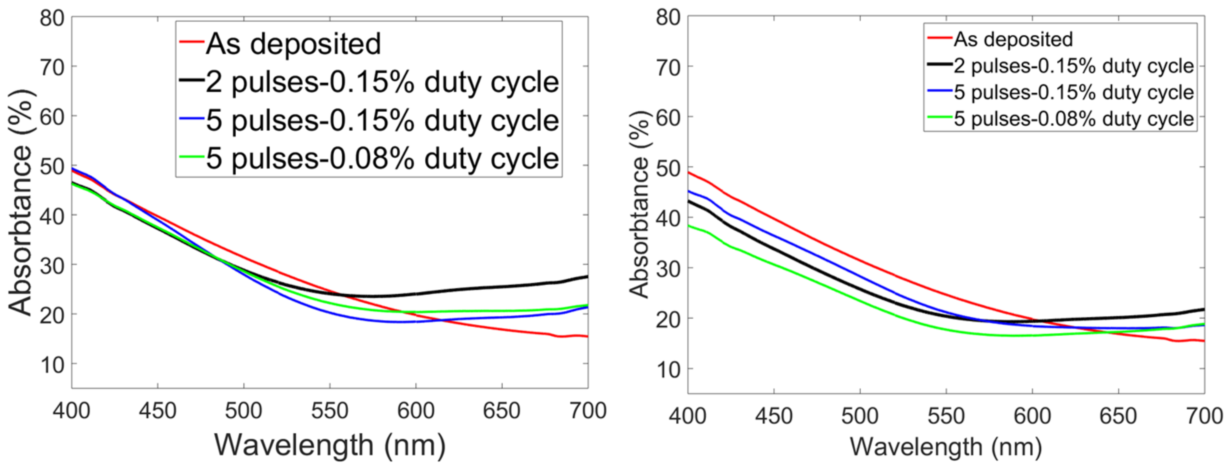

(a)

(b)

Figure S6: Optical absorbtance of the as-deposited and post-IPL films in the 400-700 nm range for IPL fluence (a) E2 (b) E3.

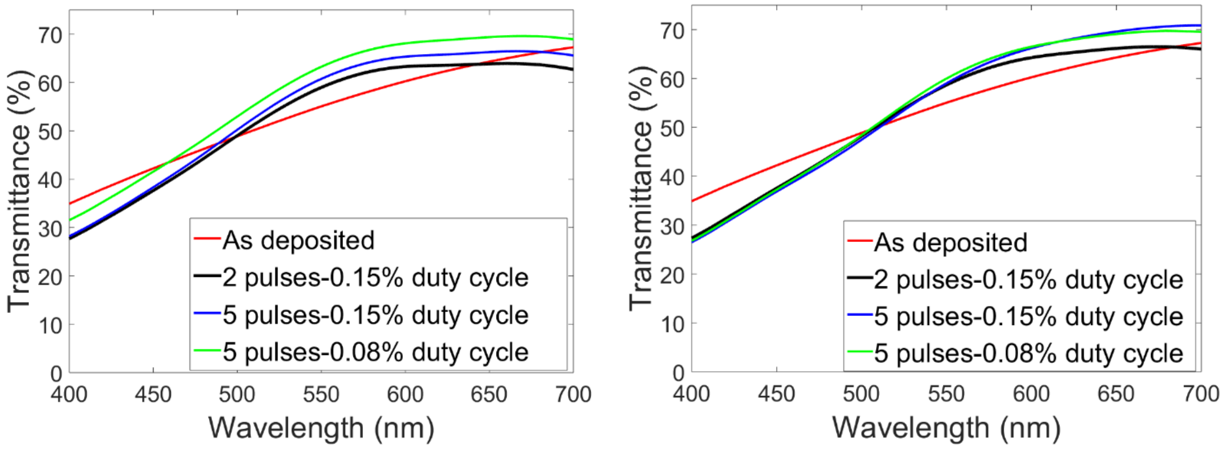

Figure S7: Optical transmittance of the as-deposited and post-IPL films in the 400-700 nm range for IPL fluence (a) E2 (b) E3.

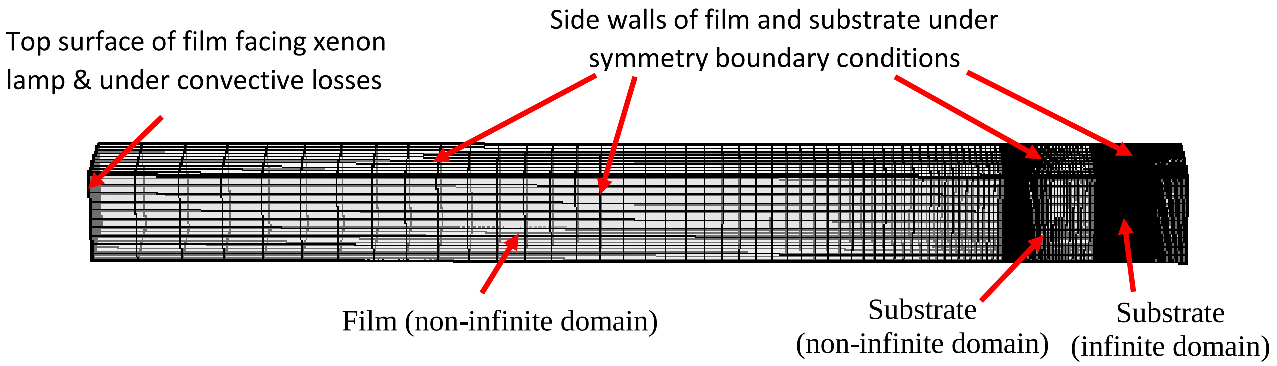

(a)

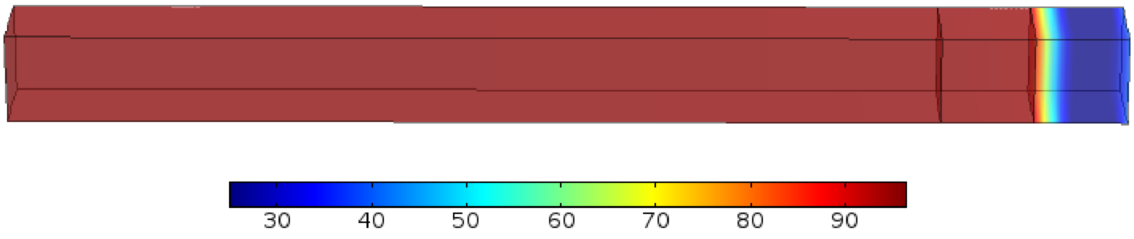

(b)

Figure S8. (a) FEA model and mesh used along with different domains in the COMSOL model (b) Example of predicted temperature profile.

References

1 Qiu, P., Zhu, Y., Qin, Y., Shi, X. & Chen, L. Electrical and thermal transports of binary copper sulfides Cu_xS with x from 1.8 to 1.96. *APL Materials* **4**, 104805, doi:10.1063/1.4953439 (2016).

2 Rumble, J. *CRC Handbook of Chemistry and Physics, 98th Edition*. (2017).
